# The neurophysiological architecture of semantic dementia: spectral dynamic causal modelling of a neurodegenerative proteinopathy

**DOI:** 10.1038/s41598-020-72847-1

**Published:** 2020-10-01

**Authors:** Elia Benhamou, Charles R. Marshall, Lucy L. Russell, Chris J. D. Hardy, Rebecca L. Bond, Harri Sivasathiaseelan, Caroline V. Greaves, Karl J. Friston, Jonathan D. Rohrer, Jason D. Warren, Adeel Razi

**Affiliations:** 1 Dementia Research Centre, UCL Queen Square Institute of Neurology, University College London, 8-11 Queen Square, London, WC1N 3AR UK; 2 Preventive Neurology Unit, Wolfson Institute of Preventive Medicine, Queen Mary University of London, London, UK; 3 Wellcome Centre for Human Neuroimaging, UCL Institute of Neurology, University College London, London, UK; 4 Turner Institute for Brain and Mental Health, School of Psychological Sciences and Monash Biomedical Imaging, Monash University, Melbourne, Australia

**Keywords:** Dementia, Magnetic resonance imaging, Neural circuits

## Abstract

The selective destruction of large-scale brain networks by pathogenic protein spread is a ubiquitous theme in neurodegenerative disease. Characterising the circuit architecture of these diseases could illuminate both their pathophysiology and the computational architecture of the cognitive processes they target. However, this is challenging using standard neuroimaging techniques. Here we addressed this issue using a novel technique—spectral dynamic causal modelling—that estimates the effective connectivity between brain regions from resting-state fMRI data. We studied patients with semantic dementia—the paradigmatic disorder of the brain system mediating world knowledge—relative to healthy older individuals. We assessed how the effective connectivity of the semantic appraisal network targeted by this disease was modulated by pathogenic protein deposition and by two key phenotypic factors, semantic impairment and behavioural disinhibition. The presence of pathogenic protein in SD weakened the normal inhibitory self-coupling of network hubs in both antero-mesial temporal lobes, with development of an abnormal excitatory fronto-temporal projection in the left cerebral hemisphere. Semantic impairment and social disinhibition were linked to a similar but more extensive profile of abnormally attenuated inhibitory self-coupling within temporal lobe regions and excitatory projections between temporal and inferior frontal regions. Our findings demonstrate that population-level dynamic causal modelling can disclose a core pathophysiological feature of proteinopathic network architecture—attenuation of inhibitory connectivity—and the key elements of distributed neuronal processing that underwrite semantic memory.

## Introduction

Normal brain operation depends on the structural and functional integrity of distributed neural networks: the disruption of these networks by pathogenic protein deposition is a fundamental theme in the pathophysiology of neurodegenerative diseases^1–4^. According to one emerging paradigm, these diseases constitute ‘molecular nexopathies’^5^: specific conjunctions between pathogenic protein and neural network characteristics, manifested in a distinctive clinico-anatomical phenotype. However, the mechanisms by which pathogenic proteins produce functional disconnections and how network damage in turn translates to the clinical phenotype remain key unsolved problems. This is partly attributable to the inherent complexity and heterogeneity of these diseases but also the difficulty of quantifying neuronal architectures—and the impact of pathogenic proteins on those architectures. Previous studies have attempted to define disease effects on macroscopic anatomical connectivity, as measured using white matter tractography^6–8^ or functional connectivity, using resting-state fMRI^9^. Functional connectivity reflects statistical dependencies between spatially remote neurophysiological events^10^, generally based on seed correlation or independent component analysis—and assessed post hoc using connectomic constructs from graph theory. Such metrics have certain limitations: they are not grounded in neuroanatomical frameworks of inter-regional (extrinsic) connectivity, cannot identify directed connections, cannot measure within-region (intrinsic) connectivity and are potentially confounded by age-related or neurodegenerative changes in neurovascular coupling. The fMRI BOLD signal comprises both neuronal and vascular components which are differentially affected by healthy aging and neurodegenerative pathologies^11,12^. Functional connectivity—the most widely studied network connectivity measure in the fMRI literature—is based on (undirected) correlations that do not distinguish or compartmentalise neuronal from cerebrovascular signalling^12^.

In contrast, dynamic causal modelling (DCM) estimates network effective connectivity—the direct (causal) effect of one neuronal population (or network element) on another^10,13^. DCM incorporates a hemodynamic model^5^ to partition the effects of neuronal interactions from Neurovascular signaling and MRI noise. A hierarchical Bayesian framework is then used to derive a model of neuronal interactions that best explain observed signal fluctuations (such as the BOLD response). By defining the direction and strength of specified connections, DCM has the potential to delineate the computational architecture of neural circuits that generate observed fMRI responses (including functional connectivity)^14^. DCM was originally designed to assess BOLD time series data with relatively small networks, limiting its applicability in neurodegenerative disease^15–17^. However, the technique of ‘spectral’ DCM, recently developed to model resting-state fMRI data, enables effective connectivity to be estimated in the spectral (frequency) domain by fitting cross-spectra rather than the underlying BOLD time series^18,19^ and is also scalable to larger networks^20,21^. Spectral DCM employs generative modelling to partition the BOLD signal into three components: a neuronal state model, describing effective connectivity; a hemodynamic state model (the well-validated, biophysical balloon model^5,22^) that characterises how neural activity is transformed into the BOLD signal; and observation or measurement noise^22^ (further mathematical details are provided in Supplementary Material). Once the generative DCM model is defined, it can be fitted to the (measured) BOLD data to furnish parameter estimates of effective connectivity that incorporate connection strength, directionality and valence (whether inhibitory or excitatory)^10,13^. Estimation of the directionality and valence of neuronal coupling (not possible using functional connectivity measures) and independence from neurovascular confounds ground spectral DCM in neurobiology and make it particularly well equipped to uncover the network architecture of neurodegenerative proteinopathies^12,14^.

Semantic dementia (SD) is the paradigmatic disorder of the human semantic memory system, characterised by selective, progressive erosion of the meanings of words, sensory objects and concepts^20–24^. Patients typically present with insidious anomic aphasia and loss of vocabulary but as the syndrome evolves, semantic impairment blights all sensory modalities and complex behavioural disturbances supervene, due to impaired understanding and evaluation of socio-emotional signals^25–27^. The SD syndrome arises from pathogenic protein deposition; principally targeting one canonical, large-scale connectivity network: the ‘semantic appraisal network’. This network is anchored in anterior temporal lobe cortex and encompasses mesial, inferior and lateral temporal and inferior frontal lobe regions in both cerebral hemispheres, albeit generally with an asymmetric, left-sided emphasis^2,4,28,29^. This leads to a highly characteristic profile of atrophy and associated white matter tract degeneration, spreading from temporal pole, fusiform gyrus and hippocampus–amygdala complex to inferior and middle temporal gyri, homologous contralateral temporal lobe regions and orbitofrontal cortex^23,28,30–32^. In the majority (> 80%) of cases, SD is underpinned by a specific histopathological subtype of pathogenic protein TDP-43 (type C) deposition^33,34^. SD therefore constitutes a neurodegenerative proteinopathy with a uniquely coherent clinical, neuroanatomical and molecular pathological signature: a cardinal ‘molecular nexopathy’^3,24^.

In the healthy brain, the intrinsic architectural features of the semantic appraisal network are well equipped to support neural processes inherent to semantic cognition. The network has been shown to have a distributed and broadly hierarchical organisation, with reciprocal interactions among participating regions^29,35–41^. An emerging synthesis of empirical data suggests that multi-modal semantic representations of objects and concepts are activated by a temporopolar cortical ‘hub’. The term ‘hub’ here refers to a region that is (in a graph theoretic sense) more strongly connected to its network than other network nodes and which (in a related, cognitive neuroscientific sense) integrates information from multiple other cortical regions and sensory processing streams^36–38,42^. The status of temporopolar cortex as a hub is well attested by an extensive body of connectivity and neuropsychological data, derived from the healthy brain and SD and other disorders^28,30,36–39,43–45^. This region integrates modality-specific representations of sensorimotor, interoceptive, affective and episodic features, based on extensive connections to temporal lobe subregions (including fusiform gyrus, amygdala and hippocampus) and extratemporal cortices^28,29,43–45^. These integrated semantic representations inform flexible and contextually appropriate, real-world behaviour via the process of controlled semantic cognition: the manipulation, evaluation and regulation of stored semantic representations by interacting top-down and bottom-up neural mechanisms instantiated in distributed anterior temporal and extra-temporal regions, including middle temporal gyrus and orbitofrontal cortex^31–33,37^. The neural circuitry of the frontal and temporal lobes is densely recurrent: this provides a substrate for local feedback loops that in turn promote the tuning of interneuronal information transfer by excitation-inhibition coupling, mediated by GABA^39^. The orchestrated balance between excitatory and inhibitory transmission is critical to normal neural circuit function and fundamentally sculpts the BOLD signal fluctuations that constitute resting-state fMRI time series^46^ . GABAergic inhibitory processes maintain efficient neural network operation by regulating the gain of neural circuit activity and stimulus reactivity and (by ‘sharpening’ circuit outputs) enable response selectivity^46,47^ . These intrinsic network electrophysiological properties—and the network connectivity they promote, as captured with fMRI—directly determine behaviour during cognitive tasks, by priming and shaping network responses to stimuli^39,46,47^ . With particular reference to semantic processing, such features would enable selective activation and predictive updating of semantic representations: processes essential to normal semantic cognition^37,39,48^.

Work in SD—the principal ‘lesion model’ of human semantic memory—has corroborated this picture. SD is associated with graded disintegration of conceptual representations: this is linked to a profound disruption of semantic network integrity (as indexed by graph theoretic parameters including reduced mean network degree, clustering coefficient and global efficiency and increased mean functional path length) with widespread abnormalities of inter-regional structural and functional connectivity, and leads to dysregulated semantic appraisal and associated abnormal behaviours^6,24,28–30,35,37,43,44,49–52^. By inference from emerging evidence in the healthy brain^39,46,47^, it is plausible that attenuation of normal inhibitory (or abnormally heightened excitatory) connections within and between the nodes of the semantic appraisal network might play a key role in the loss of network coherence and efficiency and associated semantic deficits that characterise SD. Indeed, failure of anterior temporal cortical deactivation is associated with abnormal language processing in SD^53^ while abnormally enhanced connectivity and/or reduced inhibitory GABAergic transmission within the semantic appraisal network has been linked to behavioural deficits in other neurodegenerative proteinopathies^51,54–59^. However, the underlying changes in effective connectivity wrought by SD (i.e., the crucial neural circuit characteristics of this proteinopathy and the semantic memory system it selectively targets) have not been defined.

The use of task-free, resting-state methods to define the intrinsic architecture of language networks has been strongly endorsed in SD and other neurodegenerative syndromes^60^. Such methods avoid the methodological challenges inherent in designing task-based scanning paradigms for cognitively impaired patients; moreover, task-free paradigms yield highly consistent and reproducible results and the networks these paradigms reveal map closely onto the patterns of activation during task-directed language processing^60^. With particular reference to the semantic appraisal network and SD, striking convergence of core semantic network elements has been demonstrated when task-free and task-directed connectivity patterns are compared directly, albeit with additional extra-temporal connectivity during task-based processing^38^. Furthermore, changes in resting-state network connectivity have been directly correlated with semantic deficits in SD^29,43,50,51^. Considered more broadly, semantic processing is likely to be a major constituent of the ‘default mode’ operation of the resting brain, maintaining readiness to respond appropriately to objects in the environment that impinge on homeostatic and other self-referential processes^38,45^. Taken together, this evidence suggests that resting-state connectivity techniques are a valid and informative means to identify the intrinsic network architecture that supports semantic cognition and to characterise the effects of SD on this architecture.

Here, we used spectral DCM for resting-state fMRI data to delineate the effective connectivity of the semantic appraisal network in a cohort of patients with SD of moderate severity relative to healthy older individuals. Rather than addressing a particular semantic task or deficit, our goal was to identify changes in intrinsic network architecture (evident in the resting brain) in SD that could potentially affect various active, task directed processes during semantic cognition. We targeted a small number of regions in the anterior temporal and inferior frontal lobes that have been consistently shown to be core to the neural network primarily targeted by pathogenic protein spread in SD^2,4,24,28,29,31–33,43,44,50^. Although the role of inter-hemispheric protein spread in SD is unclear^24,28^, as both cerebral hemispheres become affected in tandem with evolution of the disease, we separately explored key commissural connections linking the semantic appraisal networks in each hemisphere. Drawing on available neuropsychological, neuroanatomical and physiological evidence^37,39,50^, we hypothesised that SD would be associated with reduced network efficiency, manifest as reduced recurrent inhibition (intrinsic self-coupling) within semantic network regions and the emergence of abnormally excitatory inter-regional (extrinsic) effective connectivity. Finally, we anticipated that these effective connectivity changes would predict preeminent semantic cognitive and behavioural phenotypic features of SD.

## Results

### General characteristics of participant groups

A summary of demographic and clinical measures for the patient groups is reported in Table 1. Participant groups did not differ in age, handedness, gender nor years of education. The SD patient group differed from controls in MMSE, verbal IQ (WASI), semantic tests (graded naming test, British Picture Vocabulary Scale), verbal fluency and episodic memory for faces and words (Recognition Memory Test).

**Table 1:** Demographic, clinical and neuropsychological characteristics of participant groups.

Characteristic	Healthy controls	SD
**Demographic and clinical**		
No. (male:female)	9:11	9:5
Age (years)	67.08 (6.23)	66.29 (6.86)
Handedness (R:L)	19:1	14:0
Symptom duration (years)	N/A	6.12 (2.86)
Education (years)	16.25 (2.05)	15.69 (2.53)
MMSE (/30)	29.82 (0.39)	24.07 (6.61)*
**General intellect**		
Verbal IQ (WASI)	122 (8.79)	69 (23.66)*
Performance IQ (WASI)	122 (12.88)	116 (18.69)
**Episodic memory**		
RMT words (/30)	49 (1.20)	35 (8.05)*
RMT faces (/30)	43 (4.99)	32 (4.90)*
**Executive skills**		
WASI matrices (/32)	26 (4.35)	26 (3.84)
WMS-R digit span forward (max)	7 (0.75)	7 (0.99)
WMS-R digit span reverse (max)	6 (1.36)	5 (1.21)
D-KEFS Stroop colour naming (s)	29 (4.83)	43 (16.3)
D-KEFS Stroop word reading (s)	23 (4.40)	28 (10.72)
D-KEFS Stroop interference (s)	52 (10.04)	72 (24.64)
Trails A (s)	32 (9.31)	45 (16.41)
Trails B (s)	60 (20.45)	123 (75.20)
Letter fluency (F, 1 min)	17 (4.76)	9 (4.62)*
Category fluency (animals, 1 min)	24 (5.13)	7 (4.88)*
**Semantic skills**		
WASI vocabulary (/80)	71 (4.21)	30 (19.62)*
WASI similarities (/48)	40 (3.90)	17 (11.22)*
Graded naming test (/30)	26 (2.68)	2 (5.30)*
BPVS (/150)	148 (1.50)	78 (40.37)*
**Other skills**		
GDA (/24)	14 (5.69)	13 (4.44)
VOSP object decision (/20)	19 (1.10)	16 (2.42)

Mean (standard deviation) scores are shown unless otherwise indicated; maximum scores are shown after tests (in parentheses).

*BPVS* British Picture Vocabulary Scale; Category fluency totals for animal category and letter fluency for the letter F in 1 min, *D-KEFS* Delis Kaplan Executive System, *DS* digit span, *GDA* Graded Difficulty Arithmetic test, *GNT* Graded Naming Test, *MMSE* Mini-Mental State Examination score, *N/A* not assessed, *NART* National Adult Reading Test, *PAL* Paired Associate Learning test, *RMT* Recognition Memory Test, *SD* patient group with semantic dementia; Trails-making scores based on maximum time achievable of 2.5 min on task A and 5 min on task B, *VOSP* Visual Object and Spatial Perception Battery—Object Decision test, *WASI* Wechsler Abbreviated Scale of Intelligence, *WMS* Wechsler Memory Scale.

*Significantly different from healthy controls (based on t-tests, or chi-square tests for categorical variables).

### Accuracy of DCM model estimation

The estimation of DCM models for individual participants in both groups was excellent. Across participants, the average percentage variance-explained by DCM model estimation when fitted to the observed (cross spectra) data was 82.8% (minimum 69%; maximum 98%) for left hemisphere ROIs and 81.1% (minimum 60%; maximum 99%) for right hemisphere ROIs.

### Healthy semantic appraisal network

**Figure 1:**
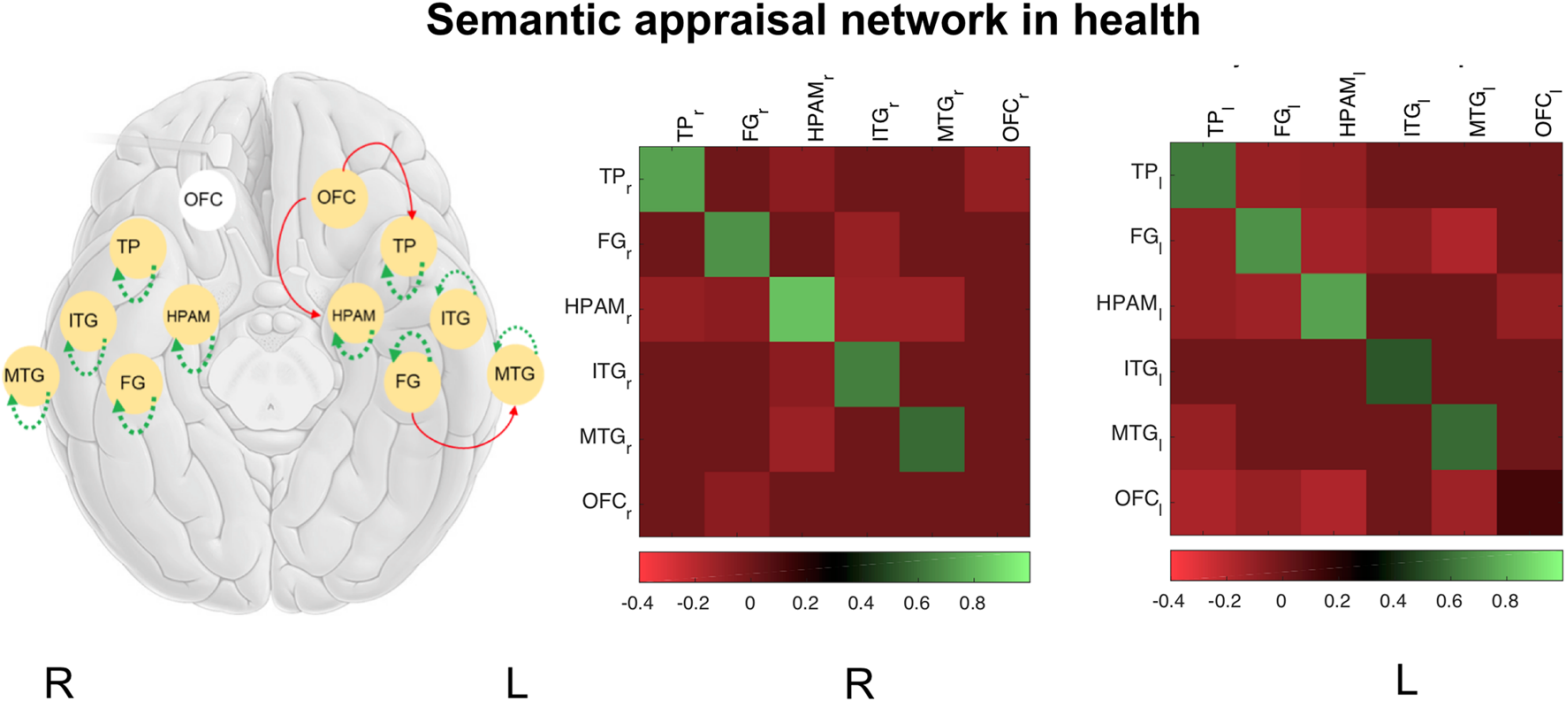
Effective connectivity of the healthy semantic appraisal network. The left panel shows a model of the network, comprising six nodes in the right (R) and left (L) cerebral hemispheres (here rendered on a cartoon view of the brain from below): *FG* fusiform gyrus, *HPAM* hippocampus–amygdala complex, *ITG* inferior temporal gyrus, *l* left, *MTG* middle temporal gyrus, *OFC* orbitofrontal cortex, *r* right, *TP* temporal pole. Gold circles indicate regions whose extrinsic connections survived Bayesian model reduction. Dashed lines indicate recurrent (intrinsic) connections within regions and solid lines indicate (extrinsic) connections between regions. Line colours code the parity of connectivity: red, inhibitory; green, excitatory (see also Table S2). The middle and right panels show the corresponding connectivity matrices for each cerebral hemisphere, the colour scale here coding connection strength (in Hz). Note: connections on the main diagonal (or self-coupling) are always inhibitory but values are log-scaled such that positive values (green; recurrent connections) indicate greater inhibition and negative values (red) less inhibition.

The healthy semantic network was characterised by strong inhibitory self-coupling within all temporal lobe regions, most marked for hippocampus–amygdala complex (Fig. 1; Supplementary Table 1), bi-hemispherically. In addition, left orbitofrontal cortex made inhibitory projections to left temporal pole and hippocampus–amygdala complex and left fusiform gyrus made an inhibitory projection to left middle temporal gyrus.

### Effect of pathogenic protein deposition

**Figure 2:**
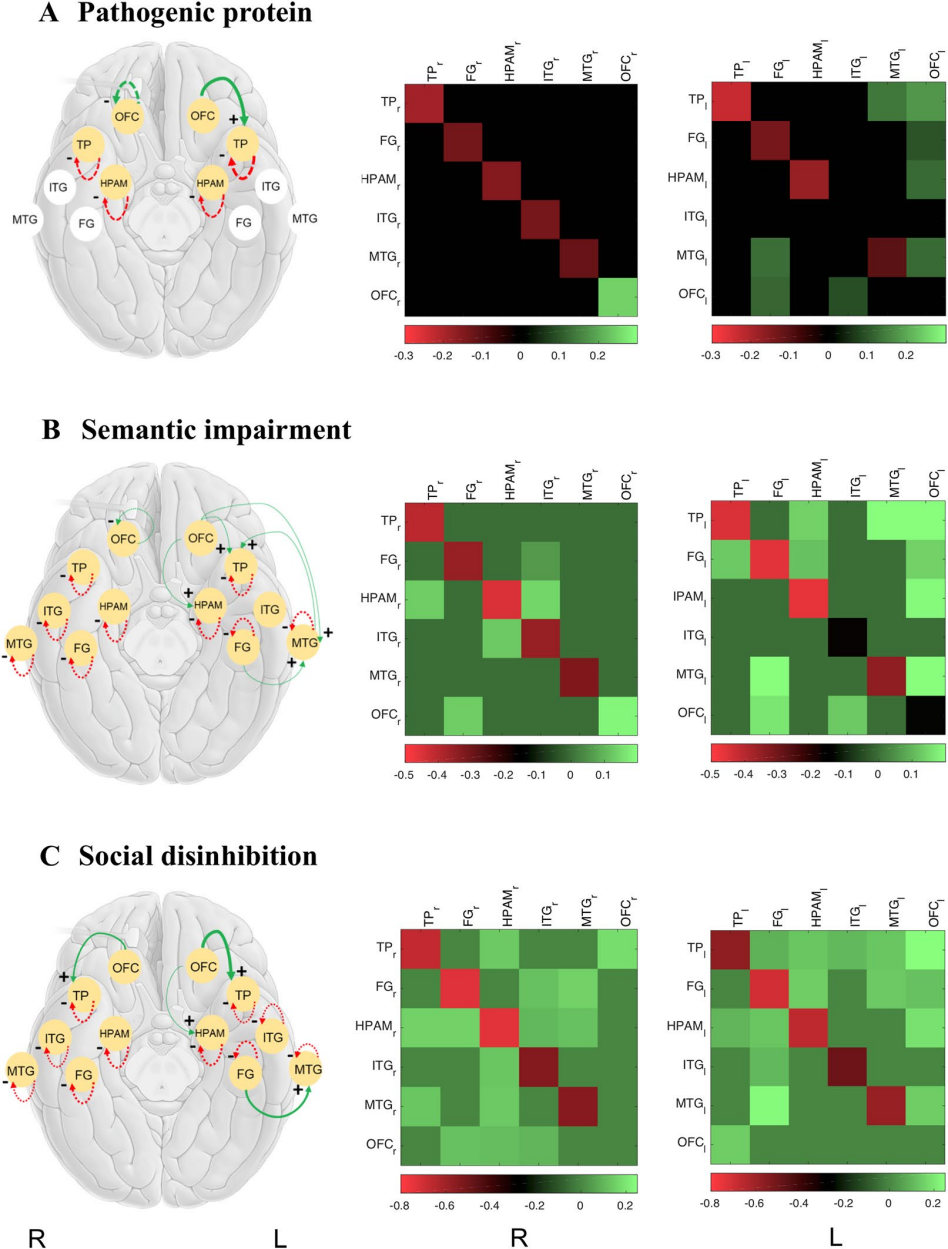
Effects of major disease factors associated with semantic dementia on effective connectivity of the semantic appraisal network. The left panels show brain cartoons representing connection changes in the right (R) and left (L) cerebral hemispheres associated with pathogenic protein deposition (**A**), semantic impairment (**B**) and disinhibited behaviour (**C**), comparing the semantic dementia group with the healthy control group (semantic dementia > controls). Gold circles code regions that show significant connectivity effects that survived Bayesian model reduction. Dashed lines indicate recurrent (intrinsic) connections within regions and solid lines indicate (extrinsic) connections between regions. Line colours code direction of connectivity changes relative to the group mean: red, decreased; green, increased. Line thickness codes the effect size; connection parity (derived by summing directional connectivity change with mean baseline connection strength) is coded as +, excitatory or **−**, inhibitory (see also Table S2). The middle and right panels show the corresponding matrices of connectivity changes for each cerebral hemisphere, the colour scale coding (log-scaled) connection strength (in Hz). Positive connectivity values (green) represent a positive change in effective connectivity with increasing score for a given disease factor while negative values (red) represent a negative change in effective connectivity with increasing score. Connectivity matrices after adjusting for regional grey matter atrophy are shown in Supplementary Figure 3 and for inter-hemispheric connections are shown in Supplementary Figure 4. *FG* fusiform gyrus, *HPAM* hippocampus–amygdala complex, *ITG* inferior temporal gyrus, *l* left cerebral hemisphere, *MTG* middle temporal gyrus, *OFC* orbitofrontal cortex, *r* right cerebral hemisphere, *TP* temporal pole.

Comparing the extrinsic and intrinsic effective connectivity profiles of the core semantic network in the SD group with the healthy control group, deposition of pathogenic protein was associated with reduced inhibitory self-coupling in the temporal pole and amygdala-hippocampus complex bi-hemispherically. In addition, there was emergence of an excitatory projection from left orbitofrontal cortex to left temporal pole; and increased inhibitory self-coupling within right orbitofrontal cortex (Fig. 2). No other alterations of intra-hemispheric or commissural (inter-hemispheric) connections were significant (Supplementary Figure 4). A model covarying for regional grey matter volume displayed the same pattern of significant results (Supplementary Table 1, Supplementary Figure 3).

### Semantic dementia phenotype

Semantic impairment (indexed by the derived composite semantic test score) was associated with widespread alterations in network connectivity (Fig. 2). These comprised reduced inhibitory self-coupling within all temporal lobe regions bi-hemispherically; emergence of excitatory projections from left orbitofrontal cortex to temporal pole, hippocampus–amygdala complex and middle temporal gyrus, from left fusiform gyrus to middle temporal gyrus and from left middle temporal gyrus to temporal pole; and increased inhibitory self-coupling within right orbitofrontal cortex. The model covarying for regional grey matter volume displayed the same pattern of significant results (Supplementary Table 1, Supplementary Figure 3).

Social disinhibition (indexed by the derived caregiver rating score; Fig. 2) was associated with reduced inhibitory self-coupling within all temporal lobe regions bi-hemispherically; and with development of excitatory projections from orbitofrontal cortex to temporal pole bi-hemispherically, from left orbitofrontal cortex to left hippocampus–amygdala complex and from left fusiform gyrus to left middle temporal gyrus. The model covarying for regional grey matter volume displayed the same pattern of significant results (Supplementary Table 1, Supplementary Figure 3).

### Connectivity drivers of disease: leave-one-out cross-validation

In a leave-one-out cross-validation of the parametric empirical Bayesian models (Table 2; Supplementary Figure 1; Supplementary Figure 2), the best predictors of diagnostic group were the excitatory projection from left orbitofrontal cortex to left temporal pole (8/14 SD patients, 8/20 healthy controls correctly classified; three SD patients, one healthy control misclassified) and the inhibitory recurrent connection of right orbitofrontal cortex (7/14 SD patients, 9/20 healthy controls correctly classified; two SD patients, no healthy controls misclassified). The best predictor of social disinhibition was the projection from left orbitofrontal cortex to left temporal pole (Table 2). The best predictors of semantic impairment were the projections from left orbitofrontal cortex to left middle temporal gyrus and from left middle temporal gyrus to left temporal pole (Table 2). Cross-validation results remained unchanged for the models covarying for regional grey matter volume.

**Table 2:** Semantic appraisal network connections as predictors of key disease factors.

Disease factor	Connection		
	Left hemisphere		Right hemisphere
	OFC to TP	OFC to MTG to TP	OFC to OFC
Pathogenic protein	Corr (df 32) = 0.55 p < 0.001	Corr (df 32) = 0.37 p = 0.001	**Corr (df 31) = 0.53 p < 0.001**
Semantic function	Corr (df 32) = 0.45 p = 0.0038	**Corr (df 32) = 0.39** **p = 0.011**	Corr (df 31) = 0.46 p = 0.0038
Social disinhibition	**Corr (df 32) = 0.67 p < 0.001**	Corr (df 32) = 0.10 p = 0.28	Corr (df 31) = 0.37 p = 0.018

This table summarises the results of leave-one-out cross validation using individual connections within the semantic appraisal network that reached the significance criterion (posterior probability > 95%) (see also Supplementary Figure 1 and Supplementary Figure 2).

Bold indicates the highest out-of-sample correlation for the connection(s) of interest between the three disease factors. Each cell specifies the Pearson’ s correlation coefficient between the observed values and the predicted values for each ‘left-out’ subject.

*MTG* middle temporal gyrus, *OFC* orbitofrontal cortex, *TP* temporal pole.

## Discussion

Here, we used spectral DCM, a novel technique for quantifying effective connectivity among distributed neuronal populations, to characterise the functional architecture of the human semantic memory system, under the impact of a specific neurodegenerative proteinopathy. The semantic appraisal network in the healthy brain at rest was revealed as a dense web of predominantly inhibitory neural connections, both recurrently within regions and between regions, with a hub in orbitofrontal cortex—projecting to two key temporal lobe regions: temporal pole and hippocampus–amygdala complex. The presence of pathogenic protein in SD weakened the normal inhibitory self-coupling of network hubs in both antero-mesial temporal lobes, with the emergence of an aberrant excitatory projections from orbitofrontal to temporal polar cortex in the more severely affected left cerebral hemisphere. Key cognitive and behavioural features of the SD phenotype—semantic impairment and social disinhibition—were linked to a similar but more extensive profile of abnormally attenuated inhibitory self-coupling within temporal lobe regions and excitatory projections between temporal and inferior frontal regions. Effective connectivity profiles remained essentially the same after adjusting for the effects of regional grey matter loss. In highlighting the network-level attenuation of intrinsic (self) inhibitory connectivity in SD, the paradigmatic disorder of semantic cognition, our findings identify both a core pathophysiological characteristic of this proteinopathy and a potentially crucial principle governing the functional anatomy of semantic memory.

Our findings reconcile previous evidence for structural and functional network disintegration in SD with computational models of the organisation of semantic cognition and its breakdown. Functional connectivity and graph theoretic analyses of resting-state fMRI data in SD have documented a generalised disruption of the physiological integrity of the semantic appraisal network, manifesting as reduced network clustering coefficient, reduced global efficiency and increased path length relative to healthy controls, emergence of subsidiary network hubs outside the canonical regions targeted by the disease and overall loss of network integrative capacity^29,43,44^. Computational models of SD have foregrounded an essential erosion of the boundaries that normally define semantic representations, traversing sensory modalities: activation of multimodal object representations becomes more dependent on irrelevant surface rather than conceptual similarities, leading simultaneously to errors of over-generalisation (e.g., a bat is classified as a bird because it has wings) and under-generalisation (e.g., an emu is not classified as a bird because it lacks wings)^37,49^. However, the neural mechanism that links network alterations to these ‘leaky’ semantic representations has not previously been defined.

Here, we have identified a candidate for this pathophysiological ‘missing link’, in the attenuation of recurrent inhibitory intrinsic connections that normally govern the semantic appraisal network in the anterior temporal lobe. Tonic inhibitory GABAergic transmission is likely to be crucial for sharpening the activation of neural representations via close coupling to phasic excitatory mechanisms and may constitute a generic principle of normal neural network function, synchronising the operation of network elements and conferring network strength, efficiency and plasticity^39,46,47,61,62^. GABA-ergic inhibitory interneurons in the more superficial cortical laminae arborise widely within cortical columns^62,63^, providing a microanatomical substrate for the normal recurrent inhibitory control over regional temporal lobe circuitry—the circuitry that is targeted by pathogenic protein deposition in SD^24^. The functional and behavioural relevance of this altered profile of intrinsic effective connectivity in SD is evidenced in the correlation with semantic impairment and social disinhibition across the temporal lobe regions sampled: this is in line with previous work associating altered resting-state functional connectivity and impaired deactivation of the semantic appraisal network with semantic impairment in SD^43,50,51,53^ and increased GABA levels in anterior temporal lobe with better semantic performance in healthy individuals^39^ as well as with evidence that attenuated inhibition may be a general mechanism of neurodegenerative pathophysiology^16,54^. It is noteworthy that the temporopolar hub—previously emphasised in models of semantic function and disruption in SD^18,19^—did not emerge as a dominant connectivity node here. It is unlikely this reflects a purely technical limitation, as review of individual MR images revealed negligible signal dropout from the temporal polar region. The connectivity of anterior temporal cortex has previously been shown to be modulated by task^38,64^: the present findings delineate the intrinsic ‘baseline’ connectivity of the semantic appraisal network, in the absence of an overt semantic task.

The profile of effective connectivity alterations in SD extended beyond local temporal lobe circuitry, additionally involving connections beyond the temporal lobe. Involvement of structural and functional connections between orbitofrontal and anterior temporal cortex—in particular, uncinate fasciculus—has been shown to be a structural signature of SD^6,29,44,65^. Functionally, this is now revealed as an abnormally enhanced excitatory communication, originating primarily in left orbitofrontal cortex. In the formulation of controlled semantic cognition^37,48,66^, inferior frontal cortex is normally engaged when dynamic ‘tuning’ of semantic representations is demanded by context and in programming an appropriate behavioural output, particularly under conditions of task difficulty or stimulus ambiguity. It is noteworthy that another core element of the ‘semantic control network’—middle temporal gyrus—was also implicated in an abnormal excitatory link to orbitofrontal cortex.

The leave-one-out validation analysis here confirmed that these abnormal orbitofrontal connections were strong predictors of pathogenic protein and phenotypic effects associated with SD. The results imply that, in a newly presenting patient, estimates of effective connectivity of efferent connections from the left orbitofrontal cortex to left temporal regions will most strongly predict both pathogenic protein deposition and the behavioural sequelae associated with SD. Given that both inferior frontal and middle temporal cortices are normally engaged during resolution of semantic ambiguity (for example, due to competing alternative resolutions or weak conceptual associations^67^)—and here showed abnormal effective connectivity correlating with behavioural measures—it is tempting to interpret the development of excitatory projections from these regions in SD as a compensatory mechanism in the face of an intrinsically degraded semantic store. A similar argument could apply to our finding of enhanced recurrent inhibitory connectivity within right orbitofrontal cortex (a region targeted relatively late by pathogenic protein deposition^32^) in the SD group. However, it is not clear that any such ‘compensatory’ mechanism would in fact be beneficial a priori. Adopting a predictive processing framework, tonic hyperactivity of ‘top-down’ inter-regional projections might establish overly precise expectations about sensory data that would tend to increase reliance on prototypical (and increasingly aberrant) object representations; particularly as any capacity for the formation of new representations and associations (updating of priors) in SD is likely to be severely compromised^37^. It has further been argued that isolated hyperconnectivity of frontal cortex may be maladaptive, reflecting reduced or abnormal feedback during the programming of behaviour^51^.

This work illuminates the pathophysiology of a poorly characterised dimension of SD; namely, its impact on complex socio-emotional behaviours. The profile of intrinsic and extrinsic effective connectivity changes linked to social disinhibition in SD was qualitatively similar to that associated with semantic impairment, albeit relatively less left-lateralised. Previous structural and tractographic studies have implicated orbitofrontal and anterior temporal cortices and their connections in the pathogenesis of disinhibition^68–71^. Studies addressing the functional connectivity of the culprit network in SD have reported altered resting-state fronto-temporal connectivity but have not addressed the mechanism of the behavioural phenotype^29,51,65^. Our findings argue for a mechanism that is at least partly in common with the pathophysiology of semantic impairment; the effective connectivity profile of social disinhibition implicating a conjunction of intrinsically degraded social conceptual representations and tonically overactive top-down control. Aberrant top-down control may promote inflexible behavioural routines: e.g., food consumption is normally heavily modulated by social mores; where relevant social knowledge is no longer accessible, the imperative to consume available food is likely to lead to faux pas. This interpretation, emphasising a deranged social lexicon rather than a release of frontal controls, is supported by neuropsychological evidence^27^.

The present findings raise a number of issues that should be addressed in further work. From a clinical perspective, spectral DCM can characterise the intrinsic functional architecture of large-scale neural networks, while discounting the potentially confounding effects of age-related haemodynamic changes. This presents an unprecedented opportunity for novel, dynamic biomarker development in neurodegenerative disease. Currently, the most widely used MRI biomarkers of neurodegeneration signal structural change (generally, brain atrophy; that is, cell death). Functional connectivity and recent variants of DCM^72,73^ which employ linear approximations to neuronal activity along with fixed and linear hemodynamic response functions are not suitable for modelling neurodegeneration, which, like ageing, differentially impacts neuronal and vascular components of the BOLD signal^11,12^. In contrast, spectral DCM, by estimating nonlinear hemodynamics allows one to separate the contribution of neuronal and vascular factors in a BOLD signal and here has revealed clinically relevant changes in neural circuit function. These circuit changes are proxies for complex clinical phenomena that are themselves difficult to measure directly. Such a dynamic, functional neuroimaging biomarker that can capture core features of the disease phenotype could be used to guide early diagnosis or to evaluate new candidate therapies, in SD and potentially other neurodegenerative proteinopathies^54,74^. However, realising this promise will require longitudinal studies employing spectral DCM in larger patient cohorts—representing a range of proteinopathies—in order to establish the sensitivity and specificity of the technique. Ultimately, this will also require histopathological or molecular correlation. DCM can detect motor changes in presymptomatic Huntington’s disease mutation carriers^75^; considered as a group, the frontotemporal dementias have a substantial genetic component that could likewise facilitate presymptomatic diagnosis^76^. This could be particularly pertinent if (as has been proposed in Alzheimer’s disease^54^) network hyperconnectivity proves to be both a marker and a driver of pathogenic protein spread in SD.

From a neurobiological perspective, the components of the semantic appraisal network as we have defined it here communicate widely with unimodal sensory cortices and higher order extra-temporal association (in particular, cingulate, insular and parietal) cortices^28–30,43,45^. These connected regions participate in other large-scale brain networks, including the salience and so-called ‘default mode’ networks^2,4^. Dynamic interactions between these networks are likely to be integral to semantic cognition, particularly when knowledge retrieval is modulated by behavioural context, self-projection or complex multidimensional objects (such as other people)^45,77,78^. However, regions (such as angular gyrus) that are strongly connected to the paradigmatic semantic network foregrounded in this study are likely to be domain-independent processors, playing a regulatory or permissive role in semantic cognition^79,80^. Our study was necessarily limited to core regions that are intrinsic to the pathogenesis of SD; the relatively small cohort size here imposed a combinatorial ‘ceiling’ on the complexity of the anatomical models that could validly be fitted. Future studies of effective connectivity in SD should address the extended connectivity architecture of semantic cognition and interactions between distributed brain networks, in larger patient cohorts that enable the fitting of more comprehensive models.

It will also be important to elucidate how effective connectivity in SD—and other neurodegenerative proteinopathies—relates to other structural and functional metrics of regional grey matter and white matter pathways and in particular, to task-related activation of vulnerable neural networks in the working brain. While semantic processing appears to be a ‘default’ resting operating characteristic of the human brain^38,45^, the activity of the semantic appraisal network is nevertheless likely to be modulated by task demands: in this regard, it may be noteworthy that temporopolar cortex, generally considered an integrative hub in current models of semantic cognition^36,37,66^, did not emerge as the most highly connected region of the semantic appraisal network in the present study nor in a previous resting-state connectivity analysis^50^. It should also be acknowledged that characterising large-scale changes in network effective connectivity is but a first step toward defining the local microcircuit properties that underpin synaptic alterations and how these mirror pathogenic protein characteristics, as anticipated by the molecular nexopathies paradigm^3^. Combining spectral DCM in these clinical populations with magnetoencephalography (which can measure changes in microcircuit laminar function) would be an attractive approach toward closing this scale gap. Additionally, however, the proper interpretation of resting-state connectivity methods when applied to complex, task-directed processes such as semantic cognition will entail a more detailed understanding of how intrinsic circuit architectural features support behavioural outputs, drawing on novel neurochemical, computational and related approaches^37,39^.

While acknowledging these caveats, the present study suggests a powerful new approach to characterising neurodegenerative proteinopathies as exemplified by SD. Semantic memory can be regarded as, essentially, a means to support neural inferences about important but unobserved states of affairs in the world at large^37^. From this perspective, uncovering the circuit architecture underpinning semantic cognition—as disclosed here by a specific proteinopathy—holds promise for our understanding of a fundamental principle of brain operation—the adaptive minimisation of prediction error—and potentially, a new computational taxonomy of neurodegenerative proteinopathies as perturbers of this process.

## Methods

### Participant characteristics

Fourteen patients fulfilling consensus criteria for SD^81^ of moderate severity (mean symptom duration 6.12 years, range 2–12 years) and 20 healthy older individuals with no history of neurological or psychiatric illness participated. Patients and healthy controls did not differ significantly in age, gender distribution or educational attainment. Neuropsychological assessment and structural brain MRI corroborated the syndromic diagnosis in all patients; no participant had radiological evidence of significant cerebrovascular damage. General clinical and neuropsychological characteristics of the participant groups are summarised in Table 1.

This study was approved by the University College London institutional ethics committee and all participants gave informed consent in accordance with the Declaration of Helsinki.

### Brain MRI acquisition and pre-processing

Resting-state functional (BOLD echoplanar imaging) and structural (T1-weighted MP-RAGE) data were acquired on a Siemens Trio 3T MRI scanner using a 32-channel phased-array head-coil. The resting-state data acquisition duration was seven minutes. Initial image preprocessing was performed using Statistical Parametric Mapping (SPM12) (https://www.fil.ion.ucl.ac.uk/spm/) and followed a standard analysis pipeline: slice timing correction, motion correction, structural and functional image co-registration, segmentation, normalisation (based on each participant's structural image) to the Montreal Neurological Institute (MNI) 152 template, and smoothing using a Gaussian kernel with a full-width half maximum 6 mm. Further details are in Supplementary Material.

Head motion is a potential confound in any study that compares patients with healthy controls. We computed the magnitude of head movements during each scan by extracting the translational and rotational motion parameters estimated during the realignment pre-processing step, and calculated the mean root-mean-squared values for translation and Euler angles for rotation. Two-sample t-tests confirmed that the healthy control and SD groups did not differ in translational or rotational motion (translational motion: controls mean (standard deviation) 0.27 (0.07) cm, SD 0.41 (0.08) cm, t = 1.25, p = 0.22; rotational motion: controls 0.005 (0.001) rad, SD 0.008 (0.001) rad, t = 1.18, p = 0.25). The range of motion was acceptable for all participants.

### Dynamic causal modelling and connectivity analyses

Effective connectivity was estimated using spectral DCM^18,19^ implemented in SPM12 r7219 (Wellcome Centre for human Neuroimaging, London, UK; code available at: https://www.fil.ion.ucl.ac.uk/spm/software/), separately for each cerebral hemisphere. Further technical background about DCM can be found in Supplementary Material.

We first defined six functional regions of interest (ROI) to represent key components of the bi-hemispheric semantic appraisal network that have been consistently implicated across studies of SD^2,4,24,28,29,33,50^: namely, the temporal pole, fusiform gyrus, hippocampus–amygdala complex, inferior temporal gyrus, middle temporal gyrus and orbitofrontal cortex (further details in Supplementary Material; see Fig. 1). For the right hemisphere, we defined six ROIs for 13 instead of 14 SD patients because of a marked signal loss restricted to medial temporal regions due to movement artefact in one case. In order to estimate the strength of within-subject, directed neural connections separately in each cerebral hemisphere (to account for hemispheric asymmetry in the distribution of disease), for each participant and hemisphere we created a fully-connected first (within-subject) level generative model of their fMRI time series—considering all possible connections among these brain regions. This model was then inverted using spectral DCM. We separately assessed a 12-ROI model to estimate inter-hemispheric connections for each participant, with the intention to look at three major commissural connections: between left and right hippocampus–amygdala complex (via anterior commissure), between left and right inferior temporal gyrus (via anterior commissure) and between left and right orbitofrontal cortex (via rostral corpus callosum)^24,28,82,83^. We performed a second (between-subjects) level analysis to estimate the group mean and effect of diagnosis for each connection. Parametric empirical Bayes routines were used to assess candidate network connectivity models at group level^84^; this procedure assesses how individual (within-subject) connections relate to group or condition means, taking account of both the expected strength of each connection and the associated uncertainty (further details in Supplementary Material). We focused on effective connections that were necessary to account for the data, with Bayesian posterior probability > 0.95 (details of all effective connections can be found in Supplementary Table 1).

We assessed three separate second-level Bayesian models, to examine changes in intrinsic and extrinsic effective connectivity (connection strength and directionality) within and between core regions of the semantic appraisal network associated with deposition of pathogenic protein and with two key phenotypic features of SD: semantic impairment and disinhibited social behaviour. The latter is a major component of the complex behavioural phenotype of SD and has been shown previously to have a structural neuroanatomical substrate encompassing various components of the targeted semantic network^25,26,51,68,69,85^; moreover, it is a relatively striking behavioural ‘signal’ that is likely to be detected by caregivers. For each participant, a ‘semantic score’ was generated using a principal component analysis of neuropsychological test scores of semantic function (details in Supplementary Table 2); a ‘social disinhibition score’ was obtained from ratings on a scale from 0 to 3 (0, absent; 1, mild; 2, moderate; 3, severe) provided by each patient’s primary caregiver and by a reliable informant for each healthy control (relevant changes in social behaviour comprised lack of adherence to social norms; e.g., inappropriate jokes or sexual comments, swearing or shouting in public). For the patient cohort, this score significantly correlated with a measure assessing disinhibition (‘acts impulsively without thinking, lacks judgment’) from the validated Frontotemporal Dementia Rating Scale (FTD-FRS)^86^ (r = 0.55, p = 0.04). For the model assessing pathogenic protein deposition, covariates were group mean and diagnostic category coded as 1 or − 1; for the models assessing phenotypic features, raw scores were scaled within the range − 1 to 1.

Bayesian model reduction was used to test all reduced models within each parent Bayesian model (assuming that a different combination of connections could exist^84^) and ‘pruning’ redundant model parameters; parameters of the best 256 pruned models (in the last Occam’s window) were averaged and weighted by their evidence (i.e. Bayesian Model Averaging) to generate final estimates of connection parameters. To identify important effects (i.e., changes in directed connectivity), we compared models (using log Bayesian model evidence to ensure the optimal balance between model complexity and accuracy) with and without each effect and calculated the posterior probability for each model, as a softmax function of the log Bayes factor. We treat effects (i.e., connection strengths and their changes) with posterior probability > 0.95 as significant for reporting purposes.

### Analysis adjusting for effects of regional grey matter loss

To determine the impact on effective connectivity profiles from regional grey matter atrophy, we assessed the effect of adjusting for the factor in a supplementary analysis. We extracted grey matter density values from segmented images (based on masks from the AAL atlas) for each of the regions included in the DCM analysis. We averaged grey matter volume over the six regions in each hemisphere and normalised by total intracranial volume. This step ensured that we looked at regionally specific atrophy and not global measures of atrophy or differences in overall brain size^17^. We included the resulting grey matter values as a covariate in all PEB models. Results for this adjusted analysis are reported in Supplementary Table 1 and Supplementary Figure 3.

### Leave-one-out validation analysis

In a leave-one-out cross-validation of the parametric empirical Bayesian models (Table 2; Supplementary Figure 1, Supplementary Figure 2), we assessed how well changes in individual network connections predicted key features of SD. Cross-validation of this kind provides out- of-sample estimates of the validity of specific connectivity strength changes for predicting the phenotype of a new patient; here, we measured the predictive posterior density over the main disease factors of interest (presence of pathogenic protein, semantic impairment, social disinhibition). Cross-validation was applied to individual connections found to be significant at group level. The connections that led to the most accurate predictions of group membership, semantic score and social disinhibition score were identified based on their out-of-sample correlation with the three factors of interest (Table 2, Supplementary Figure 1, Supplementary Figure 2).

## Supplementary information

Supplementary Information.

## Data Availability

Matlab code is available in SPM12 r7219 (https://www.fil.ion.ucl.ac.uk/spm/software/). Anonymized data will be shared on request from a qualified investigator for non-commercial research purposes within the limits of participants’ consent, and subject to institutional ethics committee approval and material transfer agreements.
