# Supplementary material for: The neurophysiological architecture of semantic dementia: spectral dynamic causal modelling of a neurodegenerative proteinopathy
Source: Sci Rep. 2020 Oct 1;10:16321. doi: 10.1038/s41598-020-72847-1 (PMC7530731; doi:10.1038/s41598-020-72847-1)
Supplement: Supplementary file 1 — Supplementary Information. [file 41598_2020_72847_MOESM1_ESM.docx]

**Supplementary Material. The neurophysiological architecture of semantic dementia: spectral dynamic causal modelling of a neurodegenerative proteinopathy, by E Benhamou et al**

Elia Benhamou^1^*, Charles R Marshall^1,2^, Lucy L Russell^1^, Chris JD Hardy^1^, Rebecca L Bond^1^, Harri Sivasathiaseelan^1^, Caroline V Greaves^1^, Karl J Friston^3^, Jonathan D Rohrer^1^,

Jason D Warren^1+^, Adeel Razi^3,4+^

^+^ these senior authors contributed equally to the work

* Corresponding Author

1 Dementia Research Centre, UCL Queen Square Institute of Neurology, University College London, London, United Kingdom

2 Preventive Neurology Unit, Wolfson Institute of Preventive Medicine, Queen Mary University of London, United Kingdom

3 Wellcome Centre for Human Neuroimaging, UCL Institute of Neurology, University College London, London, United Kingdom

4 Turner Institute for Brain and Mental Health, School of Psychological Sciences & Monash Biomedical Imaging, Monash University, Australia

Correspondence to:

Elia Benhamou

Dementia Research Centre

Institute of Neurology

8-11 Queen Square

WC1N 3AR London

United Kingdom

elia.benhamou.16@ucl.ac.uk

**Methods: Brain MR image acquisition and pre-processing**

A high-resolution anatomical image was obtained using a T1- weighted MP-RAGE structural scan (TE =2.93ms, TR = 2s, flip angle = 8°, 208 slices with 1 mm isotropic voxels) and an eyes-closed rs-fMRI sequence was obtained using BOLD contrast sensitive gradient echoplanar imaging (TE = 30ms, TR = 2.5s, flip angle = 80°, 200 volumes, 42 slices). Scans were processed using Matlab code available on-line (https://github.com/lindenmp/rs-fMRI). Pre-processing and quality control were performed as per Parkes et al.^1^. Initial image preprocessing was performed using Statistical Parametric Mapping (SPM12) (http://www.fil.ion.ucl.ac.uk/spm/) and included slice timing correction, motion correction, structural and functional image co-registration, segmentation, normalization (based on each participant's structural image) to the Montreal Neurological Institute (MNI) 152 template, and smoothing using a kernel with a full-width half maximum of 6 mm. The normalised images were interpolated to a resolution of 3 × 3 × 3 mm^3^.

**Methods: Region of interest (ROI) selection and analysis**

ROI centre coordinates for the left cerebral hemisphere were (x = -42; y = 16; z = -30) for temporal pole ^2^, (x = -34; y = -54; z = 13) for fusiform gyrus ^3^, (x = −26; y = −4; z = -26) for hippocampus-amygdala complex^4^, (x = -54; y = −2; z = -30) for inferior temporal gyrus^5^, (x =-52; y = −23; z = -9) for middle temporal gyrus^4^ and (x = -28; y = 61; z = -4) for orbitofrontal cortex^4^. Symmetrical x-coordinates were used to define ROIs in the right hemisphere (see Fig. 1). Coordinates were labelled using the AAL atlas^6^. The components of the semantic appraisal network have many additional connections beyond the network defined here but these connected regions (such as angular gyrus and posterior cingulate cortex) participate primarily in other large-scale networks (such as the so-called ‘default mode’ network). More particularly, the connected posterior parietal regions are not primary sites of pathogenic protein spread and atrophy in SD. Finally, despite such regions being strongly connected to the anterior temporal lobe, they serve very different roles in semantic cognition: in particular, the angular gyrus deactivates in a range of semantic tasks, suggesting it serves principally a regulatory or permissive role^7,8^.

Time-series were acquired by computing the principal eigenvariate of signals from voxels at specified ROI-centre coordinates using a sphere of radius 6mm for temporal pole, fusiform gyrus and orbitofrontal cortex and 4mm for other regions. Voxels were only included if they exceeded an uncorrected (whole brain) alpha-threshold of 0.05. As individual patterns and severity of regional atrophy differ between SD patients, we manually extracted each ROI by identifying the peak of functional activity closest to the centroid of the ‘original’ ROI (MNI coordinates based on previously published studies) within a masked region (from the AAL atlas). For participants in whom no supra-threshold voxels were identified, a step-wise decrease in alpha-threshold was applied until significant voxels were detected (using a lowest threshold alpha = 0.1). Visual inspection ensured that the location of each ROI was neuroanatomically accurate. Time series from the six ROIs were corrected for head motion and physiological noise. For this purpose, the nuisance regressors included the six head motion parameters, CSF (extracted from left ventricle using a 4mm sphere) and white matter (extracted from pons using a 4 mm sphere) regressors. Low-frequency signal drifts were filtered using a 128-s high-pass filter.

**Methods: Spectral dynamic causal modelling**

Dynamic causal modelling (DCM) is Bayesian framework that infers the directed (causal) connectivity among the neuronal systems – referred to as effective connectivity. A DCM technique for resting-state fMRI was recently proposed based upon a deterministic model that generates predicted cross spectra, referred to as spectral DCM. To model resting-state activity – in the absence of external stimuli – a stochastic component capturing neural fluctuations is included in the model. Mathematically, we can express the formulation of the stochastic generative model as a set of two equations. First is the neuronal state equation, namely

$\dot{x}\left( t \right)=f\left( x(t),u(t),\theta\right)+ v(t)$, (1)

and second is the observation equation, which is a static nonlinear mapping from the hidden physiological states in (1) to the observed BOLD activity and is written as:

$y\left( t \right)=h\left( x(t),\varphi\right)+ e\left( t \right),$ (2)

where $\dot{x}(t)$ is the rate of change of the neuronal states $x\left( t \right)$, $\theta$ are unknown parameters (i.e. the effective connectivity) and $v(t)$ (resp. $e(t)$) is the stochastic process – called the state noise (resp. the measurement or observation noise) – modelling the random neuronal fluctuations that drive the resting-state activity. In the observation equations, $\varphi$ are the unknown parameters of the (haemodynamic) observation function and $u(t)$ represents any exogenous (or experimental) inputs that drive the hidden states – that are usually absent in resting-state designs^9^.

Spectral DCM furnishes a constrained inversion of the stochastic model by parameterising the neuronal fluctuations$v(t)$. Spectral DCM simplifies the generative model by replacing the original timeseries with their second-order statistics (i.e., cross spectra). This means, instead of estimating time-varying hidden states, we are estimating their covariance, which is time invariant. Then, we simply need to estimate the covariance of the random fluctuations; where a scale free (power law) form for the state noise (resp. observation noise) is used – motivated from previous work on neuronal activity^10–12^ – as follows:

$g_{v}\left( \omega,\theta\right)=\alpha_{v}\omega^{-\beta_{v}}$

$g_{e}\left( \omega,\theta\right)=\alpha_{e}\omega^{-\beta_{e}}$ (3)

Here, $\left\{ \alpha,\beta\right\}\subset\theta$ are the parameters controlling the amplitudes and exponents of the spectral density of the neural fluctuations. The parameterisation of endogenous fluctuations means that the states are no longer probabilistic; hence the inversion scheme is significantly simpler, requiring estimation of only the parameters (and hyperparameters) of the model.

We used standard Bayesian model inversion (i.e. Variational Laplace) to infer the parameters of the model in (1), (2) and (3), from the observed signal$y(t)$. The description of the Bayesian model inversion procedures using Variational Laplace can be found elsewhere^13,14^.

The neuronal and hemodynamic state equations are fully described in the following papers ^14–17^ and the code can be found in spm12/Matlab (function spm_fx_fmri). The observation model is fully described in ^18^and the corresponding function in spm12/Matlab is spm_gx_fmri.

**Methods: Parametric empirical Bayes**

‘Empirical Bayes’ refers to the Bayesian inversion or fitting of hierarchical models. In hierarchical models, constraints on the posterior density over model parameters at any given level are provided by the level above. These constraints are called empirical priors because they are informed by empirical data. A hierarchical parametric empirical Bayes (PEB) model for DCM parameters was recently introduced, which represents how individual (within-subject) connections derive from the group membership^19^. This parametric random effects modelling is important because, unlike a classical test (e.g., *t*-test), it uses the full posterior density over the parameters from each participant's DCM – both the expected strength of each connection and the associated uncertainty (i.e. posterior covariance) – to inform the group-level result (i.e., group differences). Mathematically, for DCM studies with *N* participants and *M* parameters per DCM, the responses of the *i*-th participant and the distribution of the parameters over participants can be modelled as:

$y_{i}=\Gamma_{i}^{\left( 1 \right)}(\theta^{\left( 1 \right)})+ \varepsilon_{i}^{\left( 1 \right)}$

$\theta^{\left( 1 \right)}=\Gamma^{\left( 2 \right)}\left( \theta^{\left( 2 \right)} \right)+ \varepsilon^{\left( 2 \right)}$ (4)

$\theta^{\left( 2 \right)}=\eta+ \varepsilon^{\left( 3 \right)}$

where, $y_{i}$is the BOLD time series from *i-th* participant *and* $\Gamma_{i}^{\left( 1 \right)}$ is a nonlinear mapping from the parameters of a model to the predicted response $y$, which in this study was the model in Eq. S1 above. $\varepsilon_{i}^{(1)}$is independent and identically distributed observation noise (equivalent to $e\left( t \right)$ in Eq. S2). In this hierarchical form, *empirical priors* encoding second (between-subject) level effects place constraints on subject-specific parameters. The second level would be a linear model where the random effects are parameterised in terms of their precision:

$\Gamma^{\left( 2 \right)}\left( \theta^{\left( 2 \right)} \right)=(X\bigotimes W)\beta$ (5)

where, $\beta\subset\theta$ are group means or effects encoded by a design matrix with between $X$ and within-subject $W$parts. The between-subject part encodes differences among subjects or covariates such as age, while the within-subject part specifies mixtures of parameters that show random effects. We assume that the first column of the design matrix is a constant term, modelling group means and subsequent columns encode group differences.

**Supplementary Table 1.** Effective connectivity profiles of semantic appraisal network in health and semantic dementia

| Connection | **Cerebral hemisphere** | **Direction of effective connectivity change** | **Valence** | **Effect size**  (Hz) | **Effect size adj** (Hz) |
| --- | --- | --- | --- | --- | --- |
|  |  |  |  |  |  |
| Healthy semantic appraisal network | | | | | |
| *Inter-regional* |  |  |  |  |  |
| OFC to HPAM | L | NA | - | 0.18 | NA |
| FG to MTG | L | NA | - | 0.17 | NA |
| OFC to TP | L | NA | - | 0.16 | NA |
| *Recurrent*  *(intra-regional)* |  |  |  |  |  |
| HPAM to HPAM | R | NA | - | 0.83 | NA |
| HPAM to HPAM | L | NA | - | 0.74 | NA |
| TP to TP | R | NA | - | 0.74 | NA |
| FG to FG | L | NA | - | 0.70 | NA |
| FG to FG | R | NA | - | 0.70 | NA |
| ITG to ITG | R | NA | - | 0.65 | NA |
| TP to TP | L | NA | - | 0.63 | NA |
| MTG to MTG | L | NA | - | 0.59 | NA |
| MTG to MTG | R | NA | - | 0.58 | NA |
| ITG to ITG | L | NA | - | 0.54 | NA |
| Pathogenic protein deposition | | | | | |
| *Inter-regional* |  |  |  |  |  |
| OFC to TP | L | Inc | + | 0.17 | 0.17 |
| *Recurrent*  *(intra-regional)* |  |  |  |  |  |
| OFC to OFC | R | Inc | - | 0.25 | 0.28 |
| TP to TP | L | Dec | - | 0.23 | 0.18 |
| TP to TP | R | Dec | - | 0.19 | 0.14 |
| HPAM to HPAM | L | Dec | - | 0.17 | 0.10 |
| HPAM to HPAM | R | Dec | - | 0.15 | 0.09 |
| Semantic impairment | | | | | |
| *Inter-regional* |  |  |  |  |  |
| OFC to TP | L | Inc | + | 0.20 | 0.20 |
| OFC to MTG | L | Inc | + | 0.20 | 0.19 |
| FG to MTG | L | Inc | + | 0.20 | 0.21 |
| MTG to TP | L | Inc | + | 0.20 | 0.18 |
| OFC to HPAM | L | Inc | + | 0.19 | 0.17 |
| *Recurrent*  *(intra-regional)* |  |  |  |  |  |
| FG to FG | L | Dec | - | 0.46 | 0.30 |
| HPAM to HPAM | L | Dec | - | 0.46 | 0.32 |
| TP to TP | L | Dec | - | 0.45 | 0.34 |
| HPAM to HPAM | R | Dec | - | 0.45 | 0.30 |
| TP to TP | R | Dec | - | 0.40 | 0.29 |
| ITG to ITG | R | Dec | - | 0.36 | 0.26 |
| FG to FG | R | Dec | - | 0.35 | 0.24 |
| MTG to MTG | L | Dec | - | 0.34 | 0.22 |
| MTG to MTG | R | Dec | - | 0.32 | 0.23 |
| OFC to OFC | R | Inc | - | 0.18 | 0.22 |
| Disinhibited behaviour | | | | | |
| *Inter-regional* |  |  |  |  |  |
| OFC to TP | L | Inc | + | 0.28 | 0.29 |
| FG to MTG | L | Inc | + | 0.23 | 0.22 |
| OFC to HPAM | L | Inc | + | 0.19 | 0.16 |
| OFC to TP | R | Inc | + | 0.18 | 0.19 |
| *Recurrent*  *(intra-regional)* |  |  |  |  |  |
| HPAM to HPAM | R | Dec | - | 0.74 | 0.55 |
| FG to FG | R | Dec | - | 0.73 | 0.59 |
| FG to FG | L | Dec | - | 0.71 | 0.54 |
| TP to TP | R | Dec | - | 0.68 | 0.52 |
| HPAM to HPAM | L | Dec | - | 0.67 | 0.49 |
| MTG to MTG | L | Dec | - | 0.58 | 0.45 |
| TP to TP | L | Dec | - | 0.57 | 0.43 |
| MTG to MTG | R | Dec | - | 0.55 | 0.43 |
| ITG to ITG | R | Dec | - | 0.54 | 0.41 |
| ITG to ITG | L | Dec | - | 0.49 | 0.34 |

The Table summarises the effective connectivity of the semantic appraisal network in the healthy brain and changes associated with major disease factors in semantic dementia. All reported connections met our criterion of significance (>95% posterior probability); for each major disease factor, the comparisons shown are based on the contrast (semantic dementia group > healthy control group). For each connection, ‘effect size’ refers to the effective connectivity values relative to the baseline overall mean connection strength (across both the healthy control and semantic dementia groups combined); the right-most column shows the adjusted effect size (effect size adj) after covarying for regional grey matter atrophy. Connection valence was determined by summing the directional effect size with the overall mean connection strength (across the healthy and disease groups combined). +, excitatory; -, inhibitory; Inc, increased; Dec, decreased; FG, fusiform gyrus; HPAM, hippocampus-amygdala complex; Inc, increased; ITG, inferior temporal gyrus; MTG, middle temporal gyrus; NA, not applicable; OFC, orbitofrontal cortex; TP, temporal pole.

**Supplementary Table 2.** Principal component analysis parameters for assessing semantic impairment

| Variable | Component 1 | Unexplained variance |
| --- | --- | --- |
| British Picture Vocabulary Scale | 0.4590 | .08846 |
| Graded Naming Test | 0.4529 | .1125 |
| WASI Verbal IQ | 0.4545 | .1061 |

Variables here were scores on each of the three neuropsychological tests listed. These tests are known to reliably detect impairments in different dimensions of semantic function: conceptual knowledge about words and objects (verbal and visual object recognition, assessed using the Graded Naming Test and British Picture Vocabulary Scale and regulation of conceptual knowledge (subtests of the Wechsler Abbreviated Scale of Intelligence (WASI) assessing vocabulary [word definition] and similarities [associative relationships], together comprising the verbal IQ test). We generated an overall semantic score by running a principal component analysis over the three test scores; the first eigenvalue was normalised to derive a score between -1 and 1, and the rotation used was the oblique promax rotation (Kaiser off).

**Supplementary Figure 1.** Comparison of two binary classifications of participants as healthy controls (top panels) or patients (bottom panels), based on a leave-one-out cross-validation analysis. Results for two competitive predictor variables (connections) from parametric empirical Bayes models (see Supplementary Table 1) are shown. Left panels: the predictive variable was the projection from left orbitofrontal cortex (OFC) to left temporal pole (TP). Upper left: 8/20 healthy controls (black bars) were correctly classified, while three semantic dementia patients (blue bars) were wrongly assigned to the control group. Bottom left: 8/14 semantic dementia patients were correctly classified, while one healthy control participant was wrongly assigned to the patient group. Right panels: the predictive variable was the inhibitory recurrent connection of right orbitofrontal cortex. Upper right: 9/20 healthy controls were correctly classified, while two semantic dementia patients were wrongly assigned to the control group. Bottom right: 7/14 semantic dementia patients were correctly classified and no healthy controls were misclassified. Horizontal red dashed line: 95% posterior probability chosen as criterion of significance. r: Pearson’s correlation coefficient between the observed values and the predicted values for each ‘left-out’ subject.

**Supplementary Figure 2.** Comparison of true and predicted values of semantic scores (left) and behavioural disinhibition scores (right) based on leave-one-out cross validation analysis (see Table 2). Left: the predictive variable was the combination of the projections from the left middle temporal gyrus (MTG) to the left temporal pole (TP) and from the left orbitofrontal cortex (OFC) to left MTG (see Supplementary Table 1). Right: the predictive variable was the projection from the left OFC to left TP. The grey scale codes the predictive posterior probability computed at each ‘level’ of semantic and disinhibition scores (normalised between -1 and 1) for each participant; blue and red circles code observed values. Red circles refer to participants for whom the difference between predicted and observed values was >1 (5/20 healthy controls and 4/14 patients for semantic scores; 3/20 healthy controls and 3/20 patients for disinhibition scores). Subjects 1 to 20 refer to healthy control participants; subjects 21 to 34 refer to SD patients. r: Pearson’s correlation coefficient between the observed values and the predicted values for each ‘left-out’ subject.

**Supplementary Figure 3.** Comparison of effective connectivity matrices with and without covarying for grey matter atrophy. The panels show connectivity matrices changes in the left (L) and right (R) cerebral hemispheres associated with pathogenic protein deposition (A), semantic impairment (B) and disinhibited behaviour (C), comparing the semantic dementia group with the healthy control group (semantic dementia > controls). For each hemisphere, the left panels are the results reported in the main text without any covariates while right panels are the results covarying for grey matter atrophy. Positive connectivity values (green) represent a positive change in effective connectivity with increasing score for a given disease factor while negative values (red) represent a negative change in effective connectivity with increasing score. FG, fusiform gyrus; HPAM, hippocampus-amygdala complex; ITG, inferior temporal gyrus; l, left cerebral hemisphere; MTG, middle temporal gyrus; OFC, orbitofrontal cortex; r, right cerebral hemisphere; TP, temporal pole.

**Supplementary Figure 4.** Effects of major disease factors associated with semantic dementia on inter-hemispheric effective connectivity of the bi-hemispheric semantic appraisal network (12 ROIs). No significant effects were found for the three connections of interest related to anterior commissure and rostral corpus callosum: from left to right (or right to left) hippocampus-amygdala (HPAM); from left to right (or right to left) inferior temporal gyrus (ITG); from left to right (or right to left) orbitofrontal cortex (OFC). Positive connectivity values (green) represent a positive change in effective connectivity with increasing score for a given disease factor while negative values (red) represent a negative change in effective connectivity with increasing score. FG, fusiform gyrus; HPAM, hippocampus-amygdala complex; ITG, inferior temporal gyrus; l, left cerebral hemisphere; MTG, middle temporal gyrus; OFC, orbitofrontal cortex; r, right cerebral hemisphere; TP, temporal pole.

**Supplementary references**

1. Parkes, L. *et al.* Transdiagnostic variations in impulsivity and compulsivity in obsessive-compulsive disorder and gambling disorder correlate with effective connectivity in cortical-striatal-thalamic-cortical circuits. (2018) doi:10.1101/389320.

2. Collins, J. A. *et al.* Focal temporal pole atrophy and network degeneration in semantic variant primary progressive aphasia. *Brain* **140**, 457–471 (2017).

3. Chen, Y. *et al.* Brain Network for the Core Deficits of Semantic Dementia: A Neural Network Connectivity-Behavior Mapping Study. *Front. Hum. Neurosci.* **11**, (2017).

4. Seeley, W. W., Crawford, R. K., Zhou, J., Miller, B. L. & Greicius, M. D. Neurodegenerative diseases target large-scale human brain networks. *Neuron* **62**, 42–52 (2009).

5. Zhou, J., Gennatas, E. D., Kramer, J. H., Miller, B. L. & Seeley, W. W. Predicting Regional Neurodegeneration from the Healthy Brain Functional Connectome. *Neuron* **73**, 1216–1227 (2012).

6. Tzourio-Mazoyer, N. *et al.* Automated Anatomical Labeling of Activations in SPM Using a Macroscopic Anatomical Parcellation of the MNI MRI Single-Subject Brain. *NeuroImage* **15**, 273–289 (2002).

7. Humphreys, G. F., Hoffman, P., Visser, M., Binney, R. J. & Lambon Ralph, M. A. Establishing task- and modality-dependent dissociations between the semantic and default mode networks. *Proc. Natl. Acad. Sci. U. S. A.* **112**, 7857–7862 (2015).

8. Jackson, R. L., Cloutman, L. L. & Lambon Ralph, M. A. Exploring distinct default mode and semantic networks using a systematic ICA approach. *Cortex J. Devoted Study Nerv. Syst. Behav.* **113**, 279–297 (2019).

9. Friston, K. J., Kahan, J., Biswal, B. & Razi, A. A DCM for resting state fMRI. *NeuroImage* **94**, 396–407 (2014).

10. Beggs, J. M. & Plenz, D. Neuronal avalanches in neocortical circuits. *J. Neurosci. Off. J. Soc. Neurosci.* **23**, 11167–11177 (2003).

11. Stam, C. J. & de Bruin, E. A. Scale-free dynamics of global functional connectivity in the human brain. *Hum. Brain Mapp.* **22**, 97–109 (2004).

12. Shin, C.-W. & Kim, S. Self-organized criticality and scale-free properties in emergent functional neural networks. *Phys. Rev. E Stat. Nonlin. Soft Matter Phys.* **74**, 045101 (2006).

13. Razi, A. & Friston, K. J. The Connected Brain: Causality, models, and intrinsic dynamics. *IEEE Signal Process. Mag.* **33**, 14–35 (2016).

14. Friston, K. J., Harrison, L. & Penny, W. Dynamic causal modelling. *NeuroImage* **19**, 1273–1302 (2003).

15. Buxton, R. B., Wong, E. C. & Frank, L. R. Dynamics of blood flow and oxygenation changes during brain activation: the balloon model. *Magn. Reson. Med.* **39**, 855–864 (1998).

16. Stephan, K. E., Weiskopf, N., Drysdale, P. M., Robinson, P. A. & Friston, K. J. Comparing hemodynamic models with DCM. *Neuroimage* **38**, 387–401 (2007).

17. Marreiros, A. C., Kiebel, S. J. & Friston, K. J. A dynamic causal model study of neuronal population dynamics. *Neuroimage* **51**, 91–101 (2010).

18. Stephan, K. E. *et al.* Nonlinear Dynamic Causal Models for fMRI. *NeuroImage* **42**, 649–662 (2008).

19. Friston, K. J. *et al.* Bayesian model reduction and empirical Bayes for group (DCM) studies. *NeuroImage* **128**, 413–431 (2016).
